# A qualitative investigation of the supportive care experiences of people living with pancreatic and oesophagogastric cancer

**DOI:** 10.1186/s12913-022-07625-y

**Published:** 2022-02-17

**Authors:** Nadia N. Khan, Ashika Maharaj, Sue Evans, Charles Pilgrim, John Zalcberg, Wendy Brown, Paul Cashin, Daniel Croagh, Natasha Michael, Jeremy Shapiro, Kate White, Liane Ioannou

**Affiliations:** 1 Public Health and Preventive Medicine, Monash University, Melbourne, Victoria Australia; 2 Alfred Health, Melbourne, Victoria Australia; 3 Monash Health, Clayton, Victoria Australia; 4 Cabrini Health, Malvern, Victoria Australia; 5 The University of Sydney, Camperdown, New South Wales Australia

**Keywords:** Pancreatic cancer, Oesophagogastric cancer, Supportive care, Unmet needs, Symptom burden

## Abstract

**Background:**

Pancreatic and oesophagogastric (OG) cancers have a dismal prognosis and high symptom burden, with supportive care forming an integral component of the care provided to patients. This study aimed to explore the supportive care experiences of patients and caregivers living with pancreatic and OG cancers in order to identify perceived opportunities for improvement.

**Methods:**

Semi-structured individual interviews were conducted with people living with pancreatic and OG cancers, and their caregivers, across Victoria, Australia during 2020. Interviews were thematically analysed to identify common themes.

**Results:**

Forty-one participants were interviewed, including 30 patients and 11 caregivers. Three overarching themes, each with multiple sub-themes, were identified: (i) inadequate support for symptoms and issues across the cancer journey (ii) caregiver’s desire for greater support, and (iii) a multidisciplinary care team is the hallmark of a positive supportive care experience. Generally, those who had access to a cancer care coordinator and/or a palliative care team recounted more positive supportive care experiences.

**Conclusion:**

Unmet needs are prevalent across the pancreatic and OG cancer journey, with supportive care provided to varying levels of satisfaction. Greater awareness of and access to high-quality multidisciplinary support services is greatly desired by both patients with pancreatic and OG cancer and their caregivers.

**Supplementary Information:**

The online version contains supplementary material available at 10.1186/s12913-022-07625-y.

## Introduction

Supportive care forms an integral component of the overall care provided to people diagnosed with cancer, particularly those affected by cancers with low survival and high symptom burden. Pancreatic and oesophagogastric (OG) cancers fall into this category, with five-year survival rates ranging between 11 and 30%, even when detected early and treated aggressively [1]. In addition to a poor prognosis, patients experience high symptom burden and unmet needs [2] leading to heightened levels of anxiety, depression and poor quality of life [3, 4].

Care that addresses the physical, psychological, social, spiritual and information needs of patients, across the cancer care continuum, is defined as being *supportive* in nature [5]. In recent times, there has been increasing acknowledgement in clinical care of the importance of providing supportive care to patients with cancer at every step of their cancer management [6, 7]. This form of care may be provided by a range of health professionals, including (but not limited to) primary care physicians, nurses, care coordinators, dieticians, physiotherapists, occupational therapists, psychologists, speech pathologists, social workers and palliative care services.

Timely integration of supportive care in the management of patients with advanced cancer has been shown to improve quality of life, reduce symptom burden, decrease utilisation of futile aggressive end-of-life therapies as well as lead to improved survival [8, 9].

Caregivers often play a critical role in the healthcare journey of a cancer patient. An informal caregiver is defined as a person who self-nominates as providing assistance to the patient in terms of their activities of daily living, including their physical, emotional and/or spiritual needs [10]. Caregivers of cancer patients commonly experience psychological distress themselves. However, early integration of supportive care has also been shown to improve caregiver’s psychological symptoms, including levels of distress, anxiety and depression [11].

To date, there has been little exploration of the supportive care experiences of patients with pancreatic and OG cancer. In a recent study exploring the supportive care needs and use of community and allied health services amongst pancreatic and ampullary cancer patients, despite more than half of patients reporting moderate to high unmet physical and psychological needs, only 28 and 15% self-reported use of a dietician and mental health practitioner, respectively [2]. Whilst Australian Optimal Care Pathways for pancreatic and OG cancers highlight the need to provide all patients with supportive care at every step of the pathway [6, 7], there is sub-optimal understanding of the patient experience in accessing and using these services which may assist in improving access to supportive care services in the future.

Consequently, this study aimed to understand the supportive care experiences of patients, diagnosed with pancreatic and OG cancer, and their caregivers, managed in hospital sites in Victoria, Australia in order to inform potential gaps and opportunities to improve the experience and quality of supportive care provided.

## Methods

### Study design and participants

This study was designed as an inductive qualitative study. Semi-structured interviews were conducted with patients diagnosed with pancreatic and OG cancers and their informal caregivers in Victoria, Australia between April to December, 2020. Caregivers could either accompany the patient during the interview or participate in an interview individually. Caregivers were invited to participate so that they could provide support to the patient during the interview. Additionally, given the poor prognosis of these cancers, caregivers would be able to reflect on the experiences of patients who were not alive at the time of the interview or those who were too unwell to participate.

Eligibility criteria included the patient having a diagnosis of pancreatic or OG cancer, at least 4 months prior to recruitment, based on expert clinical advice that patients should have sufficient time since diagnosis to gain access to supportive care services; residence in Victoria, Australia at the time of their treatment; and be at least 18 years of age. Patients diagnosed with neuroendocrine and gastrointestinal stromal tumour types were excluded as the management differs to that of other pancreatic and OG tumour types. Non-English-speaking participants were included and interviewed in conjunction with an interpreter.

### Recruitment and consent

In order to maximise recruitment during protracted lockdown periods arising from the COVID − 19 pandemic and to ensure we captured the views of participants from varying demographic backgrounds, three recruitment methods were used: (1) principal investigators from two public and one private hospital were invited to inform their eligible patients about the study; (2) invitations (including a recruitment postcard (refer to Additional Material 1), a form for contact details and a reply-paid envelope) were sent by the Upper Gastrointestinal Cancer Registry (UGICR) to individuals, including non-English speaking individuals, participating in the registry and, (3) patients who had previously participated in research studies with the Cancer Research Program at Monash University and had expressed interest in being contacted for future research studies were also invited. Interested participants could either contact the researcher via telephone, email or the prepaid envelope to express an interest in participating. The UGICR is a clinical quality registry that collects information pertaining to the diagnosis, treatment and outcomes of patients with upper gastrointestinal cancers, and has coverage of approximately 70% of pancreatic cancer cases and 55% of OG cancer cases diagnosed across Victoria [12]. Purposeful sampling was used to recruit participants from a range of socio-demographic backgrounds.

Participants were invited to take part in a 30-45 min, semi-structured, individual interview with an experienced qualitative researcher (NNK). In order to build rapport with the participants, an introductory phone call was conducted by the interviewer with each participant prior to their interview. Participants were assured that their interview data would remain strictly confidential and not be shared with their treating teams, and that their decision to participate or not to participate would not affect their ongoing treatment.

Written informed consent was obtained by the researcher (NNK) from each participant through an electronic or hardcopy participant information and consent form (which was posted to the participant with a reply-paid envelope), depending on the participant’s preference. This study was approved by the Alfred Health Human Research Ethics Committee under the National Mutual Acceptance scheme (project 58721).

### Data collection

A semi-structured interview guide (Additional Material 2) was developed with an advisory working group comprising clinicians, allied health professionals and qualitative research methodology experts and explored the aims of this study.

All interviews were conducted via telephone or Zoom video conferencing because of an inability to interview participants face-to-face due to COVID-19 restrictions. Zoom video conferencing was offered as this would enable participants and the researcher to build rapport by being able to ‘see’ each other and also allow the researcher to observe any non-verbal cues. This also gave the participants an opportunity to partake in the interviews from the comfort and safety of their own home. An interpreter present on telephone and conference calls translated questions from the researcher and responses from non-English speaking participants. Participants were also asked to complete a short demographic information form (Additional Material 3) at the commencement of their interview. Treatment-level characteristics were extracted from the UGICR. Interviews were conducted until data saturation was achieved (i.e. no new themes emerged) in interviews with both patients and caregivers.

Interviews were audio-recorded using a digital recorder and transcribed verbatim by a professional transcription company. Participants were offered the opportunity to review their interview transcripts prior to analysis.

### Analysis

Using NVivo Version 12 software (QSR International Pty Ltd. Melbourne, Australia), interviews were analysed using the reflexive thematic analysis approach developed by Braun and Clark [13]. This form of analysis allows for the identification of patterns of meaning behind people’s experiences. This was underpinned by a phenomenological approach, using a constructivist paradigm, which recognises that the lived experiences of an individual is shaped through their social interactions, thereby giving rise to multiple, subjective truths, which are all equally valid [14].

All transcripts were analysed by NNK. In order to enhance the trustworthiness of the analysis, we utilised analyst triangulation, whereby 10% of transcripts were randomly selected and independently analysed by AM, who was not involved in the data collection process. A combination of deductive and inductive coding was performed to code for ideas anticipated based on existing literature and research questions, as well as to identify codes generated through the data. Broader themes were generated by unifying related categories, which were each made up of a cluster of similar codes [15]. A comparison of the independent coding trails and discussion between NNK and AM identified similarities and discrepancies; in the event of any discrepancies, consensus was reached by modifying or including new codes. Themes were further verified with members of the research team (SE, CP, JZ and LI) with expertise in pancreatic and OG cancer management.

Demographic, disease-related and treatment-level characteristics are presented as frequencies and percentages, with age presented as median and interquartile range.

The findings have been reported in accordance with the Standards for Reporting Qualitative Research [16].

## Results

### Demographic and clinical characteristics

A total of 41 participants were recruited between April and December, 2020 (*n* = 16 though site principal investigators, *n* = 19 through the UGICR, and *n* = 6 through previous research studies), comprising 30 patients and 11 caregivers. Eight of the 11 caregivers were interviewed with the patient they cared for (i.e. conjoint interviews), with the remaining three conducted with the caregiver only as the patient was either deceased (*n* = 1) or too ill to participate (*n* = 2). Amongst patients, more males (67%) than females (33%) participated and approximately 63% were diagnosed with OG cancer and the remaining 37% diagnosed with pancreatic cancer (Table 1). Most patients had been diagnosed at least 2 years prior to recruitment, resided in metropolitan areas (60%), were born in Australia (67%), able to converse in English (93%), educated up to year 12 (63%) and not employed at the time of the interview (77%). The majority of patients had received surgery (73% of patients with pancreatic cancer; 63% of patients with OG cancer) and/or chemotherapy (100% of patients with pancreatic cancer; 68% of patients with OG cancer). More female caregivers (91%) than males participated. The majority of caregivers resided in a metropolitan area (82%), were born in Australia (73%), unemployed at the time of the interview (55%) and educated to varying levels.

**Table 1:** Demographic, disease and treatment-related characteristics of participating patients and caregivers (*n* = 41)

	Patient (***n*** = 30) n(%)	Caregiver (***n*** = 11) n(%)
**Age in years (median (IQR))**	64.5 (59 – 73.8)	64.0 (60.5 - 72.5)
**Sex**		
Male	20 (66.7)	1 (9.1)
Female	10 (33.3)	10 (90.9)
**Cancer type**		
Pancreatic	11 (36.7)	7 (63.6)
Oesophagogastric	19 (63.3)	4 (36.4)
*Oesophageal*	*13(43.3)*	*4 (36.4)*
*GOJ*	*2 (6.7)*	*0 (0)*
*Gastric*	*4(13.3)*	*0 (0)*
**Time from diagnosis in months**		
4-9 M	10 (33.3)	7 (63.6)
10-15 M	6 (20.0)	3(27.3)
16-20 M	4 (13.3)	0 (0)
21-25 M	6 (20.0)	1 (9.1)
26-30 M	1 (3.3)	0 (0)
31-35 M	2 (6.7)	0 (0)
36 M+	1 (3.3)	0 (0)
**Location** ^**a**^		
Major City	18 (60.0)	9 (81.8)
Inner Regional	7 (23.3)	2 (18.2)
Outer Regional	5 (16.7)	0 (0)
**Born in a country other than Australia**	10 (33.3)	3 (27.3)
**Interpreter required**	2 (6.7)	0 (0)
**Education level**		
Below year 12	13 (43.3)	2 (18.2)
Year 12	6 (20.0)	2 (18.2)
Certificate or diploma	7 (23.3)	3 (27.3)
Undergraduate degree	2 (6.7)	3 (27.3)
Postgraduate degree	2 (6.7)	1 (9.1)
**Current employment status**		
Full time	5 (16.7)	1 (9.1)
Part time	1 (3.3)	3 (27.3)
Casual	1 (3.3)	1 (9.1)
No paid work	23 (76.7)	6 (54.5)
**Treatment(s) received** ^**b**^		
Pancreatic (*n* = 11)		
*Surgery*	8 (72.7)	n/a
*Chemotherapy*	11 (100.0)	n/a
*Radiotherapy*	2 (18.2)	n/a
Oesophagogastric (*n* = 19)		
*Surgery*	12 (63.2)	n/a
*Chemotherapy*	13 (68.4)	n/a
*Radiotherapy*	8 (42.1)	n/a

*Abbreviations*: *GOJ* Gastro-Oesophageal Junction, *IQR* Interquartile Range

^a^ Major city, inner and outer regional defined according to the Australian Statistical Geographical Classification Remoteness Area (ASGC-RA) 2016

^b^ %‘s do not equate to 100 as multiple response options were possible. Treatment-level data pertaining to *n* = 12 patients were derived through interviews as data for these patients were not available through the Upper Gastrointestinal Cancer Registry

### Themes

Three overarching themes, each with multiple subthemes, were identified: (i) inadequate support for symptoms and issues across the cancer journey (ii) caregiver’s desire for greater support (iii) a multidisciplinary care team is the hallmark of a positive supportive care experience. Please refer to Additional Material 4 for an overview of the themes and subthemes.

### Inadequate support for symptoms and issues across the cancer journey

#### Supportive care was not a focus at diagnosis

Patients commonly experienced a range of debilitating symptoms across all stages of their cancer journey. At diagnosis, all patients with OG cancer reported difficulty swallowing while many with pancreatic cancer recalled having jaundice. Both pancreatic and OG cancer patients recalled experiencing bloating and abdominal discomfort or pain, weight loss and nausea, accompanied by a reduced appetite at the time of diagnosis. Many of these symptoms prompted patients to seek medical advice and lead to their eventual diagnosis. However, many patients did not receive symptomatic relief during the time of diagnosis, as the focus was to quickly arrange tests and appointments with surgical, oncology and/or radiotherapy teams.

*“At that time with the shock of the whole thing and whatever, I was too busy going on. Like I said I saw my surgeon, next day scans, few days later another scan, straight away treatment” (P16, patient with OG cancer).*

*“The fact that* (the patient) *wasn’t particularly eating a great deal was not very high on anyone’s agenda, it was just all about trying to get this chemo done to try and shrink the tumour and qualify for the operation.” (P24, caregiver of patient with pancreatic cancer)*

Many patients recalled experiencing feelings of fear, shock, sadness and feeling overwhelmed at the time of diagnosis, with many hoping for greater sensitivity from clinicians when being informed of their diagnosis. Some also expressed disappointment in their cancer not being detected earlier and felt that the GP required some *“push”* to investigate further.

*“I remember going with my dad to the GP to find out answers [as to] why my dad had stomach aches … with this GP I had to go with him and say look can my dad have something done or can you forward this on…you have to push it along I think.” (P17, caregiver of patient with pancreatic cancer)*

Additionally, many patients were diagnosed and commenced treatment during the COVID-19 pandemic and discussed the challenges this posed. Interactions with health professionals were most affected during the pandemic, with many noting that their ongoing care was facilitated through telehealth appointments. This was viewed as a positive change by some as they were conscious of leaving the safety of their home in an immunocompromised state, however for others, it was a *“highly impersonal”* (P10, patient with pancreatic cancer) experience.

#### Confusing dietary information

Diet-related issues, such as difficulty eating, nausea and vomiting, as well as diarrhoea and constipation were commonly experienced across all stages of the cancer journey, with one patient recalling that he was *“vomiting about four times a week, and wasn’t eating or drinking”* (P10, patient with pancreatic cancer) during active treatment.

While all patients recalled consulting a dietician for management of diet-related issues and weight loss, this support however was provided to varying levels of satisfaction. Particularly in the post-treatment phase, only half of the participants with such needs felt satisfied with the quality of support provided by their dietician.

*“In the whole process, probably the worst thing was finding out what I should be eating…(dietician) gave me a sheet and it was all very confusing. Even the surgeon, he said, “Oh, just eat high fibre and see what you can keep down” sort of thing.” (P34, patient with OG cancer)*

Similar sentiments were echoed by non-English speaking patients who expressed (via an interpreter) that the dietary information provided by health professionals was “*a bit general*” and not reflective of the fact that “*Caucasians will have a different diet, (and that) Asian’s will have a different diet*” *(P28, patient with OG cancer).*

This lack of professional dietetic support meant that patients had to “*experiment*” (*P28, patient with OG cancer*) with their diet, “*Google*” information and “*try different things to eat*” *(P34, patient with OG cancer),* as well as rely on caregivers for additional support.

From the perspective of patients, high quality dietetic support encompassed detailed information about *“what to eat and what not to eat” (P4, patient with pancreatic cancer)*, in the form of verbal and written information that was *“more than just a pamphlet to come home with” (P23, patient with OG cancer).*

#### Inadequate pain and fatigue management and its consequences

Pain was commonly discussed as an issue across all stages of the cancer journey. GPs were commonly mentioned as support services for ongoing pain management, with very few patients mentioning the involvement of a pain specialist, palliative care physician or physiotherapist in their care. A lack of access to support services, such as physiotherapy, was also exacerbated by lockdowns imposed in response to COVID-19: “*We are in lockdown, so it’s not really easy to go to a physio.” (P20, patient with OG cancer)*.

Whilst the pain had resolved after a few weeks or months for some, for others, it continued to be an ongoing issue and was managed to varying levels of satisfaction.

*“After the first couple of days in ICU, nothing else they gave me for the pain really worked… in the end, I got resigned to the fact that I was just going to have to live with the pain.” (P27, patient with OG cancer).*

Language barriers impacted the ability of a Non-English-speaking patient to express the extent of her pain, with her caregiver noting:

*“She doesn’t understand what the doctor is trying to tell her to do and then she can’t explain where the pain is or how the pain is affecting her.” (P31, caregiver of patient with pancreatic cancer)*

Fatigue, which for many patients continued well after treatment discontinuation, also impaired the ability of patients to undertake activities of daily living.

*“I mean, I couldn't even shower myself. I was out of breath. It was terrible. I had no one helping me... house cleaning and things like that. I couldn't do that for months on end.” (P4, patient with pancreatic cancer)*

Long-term debilitating pain, combined with fatigue, had a variety of effects on the lives of patients as well as their caregivers, including financial hardship. Some patients expressed their desire to return to the workforce following treatment, however, were physically unable to do so. In these instances, patients discussed their difficulties with accessing government-funded financial aid and described this as a process which left them feeling “*horrible*” and “*disheartened*” (P18, patient with OG cancer).

Only a handful of patients, particularly those treated at private hospitals, mentioned receiving support through in-home hospice and palliative care services. A few accessed domestic supports through their local council as well as assistance from a social worker, with access to this service in particular being highlighted as an issue:

*“There’s not enough social workers, you’ve got to wait in line. Even when I was in the hospital and I asked for a social worker, there was no one available.” (P33, patient with OG cancer)*.

#### Demanding role of caregivers

A lack of professional support services meant that caregivers and extended family were most commonly identified as sources of support when patients struggled with day-to-day activities, such as cooking, cleaning, grocery shopping, transportation, showering, organising medical appointments and mediating care of the patient at home. This responsibility however, often served as challenging for caregivers*:*

*“I appealed to My Aged Care for some domestic help…I was trying to, every couple of hours, get him to eat and do things, and then medications and blood level testings, insulin, it was - it was really quite full (on)” (P13, caregiver of patient with pancreatic cancer)*

#### Lack of continuity of care

Some patients discussed a lack of continuity of care in their ongoing cancer management. For example, one caregiver recalled that the patient they cared for did not have ongoing access to a dietician because “(the) *oncology* (department) *doesn’t have the funding for a dietitian” (P6, caregiver of patient with pancreatic cancer).* Regional dwelling participants discussed the constantly evolving regional workforce and expressed their disappointment with not having a consistent GP. Additionally, patients did not know which health professionals to consult when experiencing new symptoms, issues or concerns.

*“…sometimes when I get sick, it’s like, I don’t know who I should sort of go to about my problem…am I still under the oncologist’s care even though the chemotherapy is finished? I’m not quite sure who I’m now meant to go to at this sort of stage.” (P10, patient with pancreatic cancer).*

### Caregiver’s desire for greater support

#### Guidance for appropriately caring for patient

When reflecting on the supportive care experiences of patients, caregivers often noted the lack of information and support provided to them by the health services, particularly at the time of hospital discharge following surgery. This minimal guidance created difficulties with appropriately caring for the patient at home.

*“The hospital made no attempt to explain to me what I needed to do. I had to do basically a crash course in how to use the feed, how to feed her, how to look after her and I found that very difficult in the first week or so with working out exactly what I needed to do.” (P26, caregiver of patient with pancreatic cancer)*

This issue was further exacerbated by COVID-19, with one caregiver noting that following her husband’s discharge from the intensive care unit, *“he was deposited in our car with the equipment, the IV holder and everything, and off we went home. No one actually went through discharge properly…” (P13, caregiver of patient with pancreatic cancer).*

#### Emotional support

A stark difference was observed between the emotional needs of patients compared to their caregivers. Very few patients desired professional mental health support, with most commonly citing their family, friends and community networks as strong emotional support systems and preferring to discuss their emotional and spiritual concerns with their caregiver as opposed to a health professional:

*“I’m okay with my wife at the moment. It’s better this way than talking to somebody else” (P5, patient with pancreatic cancer).*

However, despite patients having their caregiver as an *“outlet” (P10, patient with cancer)*, there was often no mental health support available for the caregivers themselves, with one caregiver noting:

*“My husband being the patient, obviously they were offering the counselling to him. If I was the doctor, I would have said, okay, you’ve got your wife, but I would have asked me the question, then, how are you coping with all this, you know?” (P6, caregiver of patient with pancreatic cancer).*

#### Financial support for regional-dwelling caregivers

Some regional dwelling caregivers also expressed a desire for access to subsidised accommodation and fuel vouchers for travelling to and from metropolitan hospitals.

*“The hospital didn’t offer any support in the way of accommodation or anything like that for partners.” (P26, caregiver of patient with pancreatic cancer)*

### A multidisciplinary care team is the hallmark of a positive supportive care experience

Most participants who were satisfied with their supportive care experiences discussed the involvement of a multidisciplinary care team including clinicians, GP, cancer care coordinator and/or palliative care service, as well as community support services. One patient expressed that he felt “*reassured*” knowing that his “*case was under control within the team*” *(P9, patient with OG cancer).*

Of those who lacked access to multidisciplinary support services, there was an explicit desire for information regarding all available services to be “*offered*” to the patient and their caregiver so that they “*can make a choice*” *(P6, caregiver of patient with pancreatic cancer)*, particularly given that patients “*don’t know what exists or doesn’t exist”,* and *“don’t know if there’s a whole range of support services out there that* (they are) *just ignorant about.” (P10, patient with pancreatic cancer)*. This was suggested by some as being mediated through the provision of a list of all available support services and key contact personnel.

#### Cancer care coordinator – a ‘one-stop-shop’

Specifically, the cancer care coordinator was commonly mentioned as an important support service. The care coordinator was described as a *“one stop shop” (P9, patient with OG cancer),* providing emotional support, plain language explanations of treatments and prognosis, information about other support services, as well as assistance with organising appointments with various specialists. This service provided patients and caregivers with a sense of *“relief” (P18, patient with OG cancer),* particularly at a time when most felt they were in *“such a shock” (P1, patient with OG cancer).*

*“Throughout the process, those sort of questions of fear and what it actually meant and whether I could survive this, I remember having conversations with my liaison person, from the surgical team.” (P9, patient with OG cancer)*

Strikingly, those who did not discuss the involvement of a cancer care coordinator in their cancer journey expressed a desire for access to a health professional who provides ongoing support across the cancer care continuum.

*“It would have been nice if there'd been somebody that would ring me and say how are you going? Is there anything I can do for you?” (P4, patient with pancreatic cancer)*

*“You could allocate a worker to call once a week and just ask them how they're going. Because really, unless it was sort of flagged at the clinic or you raised it, you really felt like you were on your own.” (P3, caregiver of patient with OG cancer)*

#### Palliative care – ‘a form of companionship’

Of the few patients who accessed palliative care services, all spoke highly of the support provided and felt that it was an integral component of their overall cancer management, with one participant expressing that he initially *“misunderstood the value of these* (palliative care) *nurses, which is terrific, because they just pop in and see how you’re going...it’s sort of a form of companionship…” (P19, patient with OG cancer).*

Palliative care was also viewed as a support service for caregivers:

*“It’s important for me because if I’m concerned about something, I have someone else to refer to…we can talk for an hour, which you can’t really do with your GP.” (P20, caregiver of patient with OG cancer).*

#### Community support services

Patients and caregivers also discussed the role of community support services such as Pancare and Cancer Council in providing plain language information and emotional support, and directing them to support groups. However, knowledge of these community services was often self-sought, with a desire expressed for information of these services to be offered at diagnosis: *“I found Pancare on the internet…that was something the hospital could have done. They could have given information on that early in the piece actually.” (P26, caregiver of patient with pancreatic cancer).*

## Discussion

This qualitative study sheds light on the lived supportive care experiences of people affected by pancreatic and OG cancers. The greatest number of supportive care needs included, diet-related issues, weight loss, pain and difficulties with activities of daily living, with system-level barriers to adequate supportive care provision being evident. Multidisciplinary care teams were identified as an important hallmark of a positive supportive care experience. Caregivers played a critical role in supporting patients through their cancer journey and highlighted a need to be supported themselves, particularly for their emotional health and wellbeing.

With regards to the supportive care needs experienced by patients with pancreatic and OG cancer, most are consistent with existing literature. Larger-scale studies which have evaluated unmet needs in pancreatic cancer patients using The Supportive Care Needs Survey, have documented the domain of physical/daily living as being a commonly reported unmet need [2, 17]. This aligns with our findings whereby participants commonly cited treatment-related side effects, pain, fatigue and difficulty managing day-to-day activities as issues across their cancer journey.

The dietetic support experiences of patients in this study echo a previous qualitative study in which pancreatic cancer patients were dissatisfied with the level and quality of dietetic support offered [18]. Issues related to nutrition and diarrhoea are commonly experienced by OG cancer patients [19], which was also evident in this study. Given these cancers affect the gastrointestinal system, provision of high-quality dietetic support which addresses functional problems is critical. According to our participants, the dietetic support offered ought to be detailed and personally-tailored to their individual needs and cultural backgrounds.

Despite pain being a commonly reported issue in pancreatic cancer [20] and reported by many patients in this study as an ongoing problem, very few were referred to a pain specialist. Severe and enduring pain in the setting of pancreatic cancer has been associated with poor prognosis [21], therefore making it critical for patients to be provided with timely pain management support.

Similarly, fatigue was noted as an ongoing issue for many patients with pancreatic and OG cancers, impairing their ability to undertake activities of daily living as well as return to work. Concerningly, most patients did not receive any professional support for managing their fatigue. A variety of approaches, such as physical exercise, psychosocial support, complementary and alternative therapies, as well as pharmacological interventions have been shown to reduce cancer related fatigue [22–25], further highlighting the benefits of multidisciplinary supportive care.

Of those who recounted positive supportive care experiences, most recalled being managed by a multidisciplinary team of health professionals, with GPs, cancer care coordinators and palliative care teams often being cited as important sources of support. The role of cancer care coordinators in improving patient experiences is well documented, with studies showing a decrease in time to treatment [26, 27], improved patient satisfaction and more streamlined care [28] when a cancer care coordinator is involved. Similarly, a key message which came across in this study was the desire by both patients and caregivers to have access to a liaison person, who would regularly touch base with them, enquire about their needs and offer ongoing support. The availability of a centralised cancer care coordinator could assist in addressing this need.

Both patients and caregivers in our study who were linked in with a palliative care service felt well supported throughout their cancer journey. According to existing literature, patients with early access to palliative care services report better quality of supportive care as measured by the Cancer Quality Assessing Symptoms Side Effects and Indicators of Supportive Treatment [29]. When comparing communication between patients and oncologists and patients and palliative care physicians, the latter tend to assess patient understanding of prognosis and treatment, patient coping and caregiver’s needs and experiences to a greater extent compared to discussions with an oncologist [30]. Additionally, palliative and pain specialists can greatly assist with pain management. In our study, very few patients however recalled being linked with a palliative care service, suggesting the need for greater access to palliative care in this population, earlier referrals for all patients (regardless of prognosis), as well as initiatives to reduce the stigma often associated with palliative care.

In this study, patients and caregivers expressed a strong desire to be informed of all available support services, enabling them to make an informed choice of accessing support, when required. In order to enable identification of specific supportive care needs, models of care centred around measurement of patient reported outcomes for symptom monitoring [31] may be beneficial for patients. However, as expressed by participants in our study, it is important that when a need arises, the appropriate support service is accessible and of high quality. Consequently, appropriate health policies should be in place which enable access for all pancreatic and OG cancer patients to important services such as care-coordination, dietetics, psycho-oncology, social work, physiotherapy, pain management and palliative care.

This study further highlighted the critical role that caregivers play in managing the care of patients at home and also providing emotional support. Adjusting to and navigating in-home care for patients can often be stressful and tiring for caregivers, particularly at an emotionally vulnerable time when many are preparing for the death of the person in their care [32]. Coupled with existing literature, our finding that many patients did not feel a need to access professional mental health services as this support was often sought through family, friends and community networks [2, 33], further highlights the role of caregivers in attending to the emotional health and wellbeing needs of the person they care for. However, this can pose a challenge for caregivers with many in this study expressing a strong desire for psychological support themselves. Similar to a previous study, most caregivers reported little attempt by health professionals to offer them support services [34]. It is important that the needs of caregivers are identified, potentially through the collection of caregiver-reported outcomes [35]. However, adequate policies should be in place which enable cancer caregivers access to affordable and on-going support services, which meet the changing needs of caregivers across the care continuum [35]*.*

Given the timing of this study, it provides a unique perspective into the impact of the COVID-19 pandemic on the supportive care experiences of vulnerable cancer patients. As existing studies have noted delays in cancer treatment due to the pandemic [36–38], similarly, in this study it appeared that access to supportive care services, such as physiotherapy, were negatively impacted by the lockdown restrictions imposed at the time. A lack of timely access to appropriate support services, particularly in such low-survival cancers, may negatively impact the health outcomes of patients, as well as create additional burden for caregivers.

### Strengths and limitations

A major strength of this study lies in its ability to capture the first-hand, supportive care experiences from patients and caregivers across metropolitan and regional areas and the inclusion of insights from those from English and non-English speaking backgrounds. We employed researcher triangulation, whereby a proportion of transcripts were independently analysed by two researchers in order to increase the trustworthiness of our findings. Additionally, by utilising a consumer-centred approach, this study has been able to identify clear areas of supportive care provision requiring improvement, as well as suggestions of how these can be improved. Consequently, future work should focus on understanding the barriers to supportive care provision from the health system perspective, as well as co-designing supportive care interventions and resources with patients and caregivers, which meet their evolving needs.

Despite these strengths, there are a number of noteworthy limitations. Given we interviewed a small proportion of participants, we do not know whether our findings are generalisable to the larger pancreatic and OG cancer population. More male patients than females participated in this study, however this was expected given that males are more commonly diagnosed with pancreatic and OG cancers than females [1]. Amongst the included cancer types, there was a low representation of gastric cancer patients. However, we anticipate that the needs and experiences of gastric cancer patients would be similar to those of oesophageal cancer patients. The sample was weighted towards those patients who underwent surgery and had survived for at least 2 years. Additionally, given many patients were diagnosed and treated during the COVID-19 pandemic, the supportive care experiences of patients may have been impacted by the lockdowns and restrictions imposed in Victoria throughout 2020 and may not necessarily be representative of experiences associated with usual care.

## Conclusion

This qualitative investigation sheds light on the supportive care needs and experiences of people with pancreatic and OG cancer. Our findings suggest that unmet needs are prevalent across the cancer journey, and greater awareness of and access to high-quality multidisciplinary support services is greatly desired by people living with pancreatic and OG cancers and their caregivers, across all stages of their cancer journey. These findings serve as a foundation upon which future supportive care interventions and policy-related decisions around resource allocation can be based.

## Supplementary Information

**Additional file 1.**

**Additional file 2.**

**Additional file 3.**

**Additional file 4.**

## Data Availability

Data are not publicly available in order to preserve the confidentially of participants, however, are available from the corresponding author on reasonable request.
