# Supplementary material for: A qualitative investigation of the supportive care experiences of people living with pancreatic and oesophagogastric cancer
Source: BMC Health Serv Res. 2022 Feb 17;22:213. doi: 10.1186/s12913-022-07625-y (PMC8851733; doi:10.1186/s12913-022-07625-y)
Supplement: Supplementary file 1 — Additional file 1. [file 12913_2022_7625_MOESM1_ESM.pdf]

Have you been diagnosed with or do you care for someone who has been diagnosed with pancreatic, oesophageal or stomach cancer?

Are you interested in helping to improve the quality of care and support provided to patients diagnosed with pancreatic, oesophageal and stomach cancers? If yes, we would be interested to hear from you. **Please turn over for more information.**

The Project has been approved by the Alfred Hospital Ethics Committee <58721>

MONASH University | PROpatient

PROOF

Version 2 | 10 07 2020

Researchers at Monash University are interested in helping to improve the care that is provided to patients diagnosed with pancreatic, oesophageal and stomach cancers for managing their symptoms and cancer-related issues. We are interested in speaking to patients and caregivers.

Participation in this research study involves attending one, 30-45 minutes individual interview, to discuss your experiences with receiving support for managing your symptoms and cancer-related issues. The interview can be arranged through Zoom Video Conferencing or over the telephone.

If English is not your preferred language, an interpreter can be arranged.

If you are interested in participating, please contact:

Nadia Khan

(03) 9903 0028 | nadia.khan@monash.edu

If you would like one of the researchers to contact you, please complete the attached form and return using the reply-paid envelope.

PROOF
