# Supplementary material for: A qualitative investigation of the supportive care experiences of people living with pancreatic and oesophagogastric cancer
Source: BMC Health Serv Res. 2022 Feb 17;22:213. doi: 10.1186/s12913-022-07625-y (PMC8851733; doi:10.1186/s12913-022-07625-y)
Supplement: Supplementary file 2 — Additional file 2. [file 12913_2022_7625_MOESM2_ESM.docx]

**A qualitative investigation of the supportive care experiences of people living with pancreatic and oesophagogastric cancer**

Nadia N Khan^1^, Ashika Maharaj^1^, Sue Evans^1^, Charles Pilgrim^2^, John Zalcberg^1^, Wendy Brown^2^, Paul Cashin^3^, Daniel Croagh^3^, Natasha Michael^4^, Jeremy Shapiro^4^, Kate White^5^ and Liane Ioannou^1^

Affiliations

1. Public Health and Preventive Medicine, Monash University, Melbourne, Victoria, Australia
2. Alfred Health, Melbourne, Victoria, Australia
3. Monash Health, Clayton, Victoria, Australia
4. Cabrini Health, Malvern, Victoria, Australia
5. The University of Sydney, New South Wales, Australia

Corresponding author: Dr Liane Ioannou, [liane.ioannou@monash.edu](mailto:liane.ioannou@monash.edu)

# Patient Interview Guide

Hi, thank-you so much for volunteering your time today. My name is Nadia and I am a PhD student from Monash University.

Have you had a chance to go through the Participant Information and Consent Form and sign it? Did you have any questions about the study before we begin?

Before I begin the interview, I will summarise some key points and explain why we are conducting this study:

- Patients diagnosed with pancreatic, oesophageal and stomach cancers tend to experience a range of symptoms, issues and concerns that can affect their day to day life. These symptoms or issues may be physical, psychological, social or even spiritual in nature. Common symptoms or issues include:
- Weight loss
- Pain
- Distress or fear or issues coping
- Difficulty performing day to day activities
- Difficulty swallowing or pain when swallowing

*Oesophagogastric cancer only*

- Nausea
- Financial difficulties
- Support is available to help patients manage these symptoms, issues and concerns.
- The purpose of the interview today is to understand your experiences with receiving help and support for managing your symptoms and other cancer-related issues and concerns. By gaining an understanding of this, we will hopefully be able to better inform the type of support that patients receive in the future.

I will be recording this discussion and taking notes to help me remember what you said. Your answers and personal information will be strictly confidential and any information published will be made anonymous. Your participation is completely voluntary and you can choose not to answer questions, or to leave at any point.

Before we begin, do you have any questions? Are you willing to proceed with the interview?

|  | **QUESTION** | **PROMPT** |
| --- | --- | --- |
| 1 | If you don’t mind, could you please tell me the story of your diagnosis? | When did you start noticing that something wasn’t quite right?  What were some of the signs or symptoms that you experienced? |
| 2 | Can you please tell me about your experience with receiving any help or support from your doctor for managing your symptoms or issues? | Did your doctor refer you to another type of health professional or service? If yes, who or what? |
| 3 | Can you please tell me about your experience with seeking any help for managing your symptoms or issues? | If *patient sought help,* who did you seek help from? (e.g. a health professional/support group/family)  If *patient did not seek help*, was there any particular reason why you didn’t seek help? |
| 4 | Can you please tell me about your experience with seeing a <*insert name of supportive care HP/Service*>? | What type of support or information were you offered?  Did you find it useful? Why or why not?  If *patient did not attend appointment,* was there any particular reason why you didn’t see <*insert name of supportive care HP/service*>? |
| *Depending on which stage the patient is at along their cancer journey, repeat question’s 1-4 for each of the following stages, as appropriate: pre-treatment, treatment, post-treatment.*  *PROBING QUESTION:* Have you undergone any treatment? | | |
| 5 | Was there any point during or after your diagnosis when you would have liked for your doctor to refer you to another health professional or support service to help manage your symptoms or issues? | When would some support have been most useful?  What type of support or information would have been most useful?  Who would you have liked to receive this support from? |

That is all the questions I had for you today. Thank you so much for your time and for sharing your experiences. I really appreciate it. Before we end the interview, do you have any final comments you would like to make?

<*Offer patient a copy of Cancer Council Victoria’s Information and Support Services pamphlet. If patient would like more information on supportive care services, direct to WeCan website* [*https://wecan.org.au/*](https://wecan.org.au/) >

# Caregiver Interview Guide

Hi, thank-you so much for volunteering your time today. My name is Nadia and I am a PhD student from Monash University.

Have you had a chance to go through the Participant Information and Consent Form and sign it? Did you have any questions about the study before we begin?

Before I begin the interview, I will summarise some key points and explain why we are conducting this study:

- Patients diagnosed with pancreatic, oesophageal and stomach cancers tend to experience a range of symptoms, issues and concerns that can affect their day to day life. These symptoms or issues may be physical, psychological, social or even spiritual in nature. Common symptoms or issues include:
- Weight loss
- Pain
- Distress or fear or issues coping
- Difficulty performing day to day activities
- Difficulty swallowing or pain when swallowing

*Oesophagogastric cancer only*

- Nausea
- Financial difficulties
- Support is available to help patients manage these symptoms, issues and concerns.
- The purpose of the interview today is to understand your experiences with receiving help and support for managing <*insert name of patient*> symptoms and other cancer-related issues and concerns. By gaining an understanding of this, we will hopefully be able to better inform the type of support that patients receive in the future.

I will be recording this discussion and taking notes to help me remember what you said. Your answers and personal information will be strictly confidential and any information published will be made anonymous. Your participation is completely voluntary and you can choose not to answer questions, or to leave at any point.

Before we begin, do you have any questions? Are you willing to proceed with the interview?

|  | **QUESTION** | **PROMPT** |
| --- | --- | --- |
| 1 | If you don’t mind, could you please tell me the story of <*insert name of patient*> diagnosis? | When did he/she start noticing that something wasn’t quite right?  What were some of the signs or symptoms that he/she experienced? |
| 2 | Can you please tell me about your experience with receiving any help or support from your doctor for managing <*insert name of patient*> symptoms or issues? | Did your doctor refer <*insert name of patient*> to another type of health professional or service? If yes, who or what? |
| 3 | Can you please tell me about <*insert name of patient*> experience with seeking any help for managing his/her symptoms or issues? | If *patient sought help,* who did he/she seek help from? (e.g. a health professional/support group/family)  If *patient did not seek help*, was there any particular reason why he/she didn’t seek help? |
| 4 | Can you please tell me about <*insert name of patient*> experience with seeing a <*insert name of supportive care HP/Service*>? | What type of support or information was he/she offered?  Did he/she find it useful? Why or why not?  If *patient did not attend appointment,* was there any particular reason why he/she didn’t see <*insert name of supportive care HP/service*>? |
| *Depending on which stage the patient is at along their cancer journey, repeat question’s 1-4 for each of the following stages, as appropriate: pre-treatment, treatment, post-treatment.*  *PROBING QUESTION:* Has <*insert name of patient*> undergone any treatment? | | |
| 5 | Was there any point during or after <*insert name of patient*> diagnosis when you would have liked for the doctor to refer <*insert name of patient*> to another health professional or support service to help manage his/her symptoms or issues? | When would some support have been most useful?  What type of support or information would have been most useful?  Who would <*insert name of patient*> have liked to receive this support from? |

That is all the questions I had for you today. Thank you so much for your time and for sharing your experiences. I really appreciate it. Before we end the interview, do you have any final comments you would like to make?

<*Offer caregiver a copy of Cancer Council Victoria’s Information and Support Services pamphlet. If caregiver would like more information on supportive care services, direct to WeCan website* [*https://wecan.org.au/*](https://wecan.org.au/) >
