# Supplementary material for: A qualitative investigation of the supportive care experiences of people living with pancreatic and oesophagogastric cancer
Source: BMC Health Serv Res. 2022 Feb 17;22:213. doi: 10.1186/s12913-022-07625-y (PMC8851733; doi:10.1186/s12913-022-07625-y)
Supplement: Supplementary file 3 — Additional file 3. [file 12913_2022_7625_MOESM3_ESM.docx]

**A qualitative investigation of the supportive care experiences of people living with pancreatic and oesophagogastric cancer**

Nadia N Khan^1^, Ashika Maharaj^1^, Sue Evans^1^, Charles Pilgrim^2^, John Zalcberg^1^, Wendy Brown^2^, Paul Cashin^3^, Daniel Croagh^3^, Natasha Michael^4^, Jeremy Shapiro^4^, Kate White^5^ and Liane Ioannou^1^

Affiliations

1. Public Health and Preventive Medicine, Monash University, Melbourne, Victoria, Australia
2. Alfred Health, Melbourne, Victoria, Australia
3. Monash Health, Clayton, Victoria, Australia
4. Cabrini Health, Malvern, Victoria, Australia
5. The University of Sydney, New South Wales, Australia

Corresponding author: Dr Liane Ioannou, [liane.ioannou@monash.edu](mailto:liane.ioannou@monash.edu)

**Demographic information form**

| **Participant ID: _ _**  **Date: _ _ / _ _ / _ _ _ _** | |
| --- | --- |
| What is your age? |  |
| What is your sex? (*please tick*) | □ Female  □ Male  □ Other |
| **PATIENT:** Which of the following cancer types have you been diagnosed with (*please tick*):  **CAREGIVER**: Which of the following cancer types has the person you care for been diagnosed with (*please tick*): | □ Pancreatic cancer  □ Oesophageal cancer  □ Stomach (or gastric) cancer |
| **PATIENT:** How long ago were you diagnosed with pancreatic, oesophageal or gastric cancer?  **CAREGIVER:** How long ago was the person you care for diagnosed with pancreatic, oesophageal or gastric cancer? |  |
| Which country were you born in? (*please tick*) | □ Australia  □ Other (*please specify below*) ________________________________ |
| If you were born in a country other than Australia, how many years have you resided in Australia? |  |
| What is your postcode of residence? | _ _ _ _ |
| Are you of Aboriginal or Torres Strait Islander descent? (*please tick*) | □ Yes  □ No  □ Prefer not to answer |
| What is your highest level of education? (*please tick*) | □ Below year 12  □ Year 12  □ Certificate or diploma  □ Undergraduate degree  □ Postgraduate degree |
| Do you currently work: (*please tick*) | □ Full time  □ Part time  □ Casual  □ No paid work |
