# Supplementary material for: A qualitative investigation of the supportive care experiences of people living with pancreatic and oesophagogastric cancer
Source: BMC Health Serv Res. 2022 Feb 17;22:213. doi: 10.1186/s12913-022-07625-y (PMC8851733; doi:10.1186/s12913-022-07625-y)
Supplement: Supplementary file 4 — Additional file 4. [file 12913_2022_7625_MOESM4_ESM.docx]

**A qualitative investigation of the supportive care experiences of people living with pancreatic and oesophagogastric cancer**

Nadia N Khan^1^, Ashika Maharaj^1^, Sue Evans^1^, Charles Pilgrim^2^, John Zalcberg^1^, Wendy Brown^2^, Paul Cashin^3^, Daniel Croagh^3^, Natasha Michael^4^, Jeremy Shapiro^4^, Kate White^5^ and Liane Ioannou^1^

Affiliations

1. Public Health and Preventive Medicine, Monash University, Melbourne, Victoria, Australia
2. Alfred Health, Melbourne, Victoria, Australia
3. Monash Health, Clayton, Victoria, Australia
4. Cabrini Health, Malvern, Victoria, Australia
5. The University of Sydney, New South Wales, Australia

Corresponding author: Dr Liane Ioannou, [liane.ioannou@monash.edu](mailto:liane.ioannou@monash.edu)

**Supplemental table 4: Overview of themes and sub-themes**

| Themes | Subtheme(s) |  |
| --- | --- | --- |
| Inadequate support for symptoms and issues across the cancer journey | Supportive care was not a focus at diagnosis | *“The fact that (the patient) wasn’t particularly eating a great deal was not very high on anyone’s agenda, it was just all about trying to get this chemo done to try and shrink the tumour and qualify for the operation.”* (P24, caregiver of patient with pancreatic cancer) |
|  | Confusing dietary information | *“In the whole process, probably the worst thing was finding out what I should be eating…(dietician) gave me a sheet and it was all very confusing. Even the surgeon, he said, “Oh, just eat high fibre and see what you can keep down” sort of thing… So, Mr Google came in quite handy.”* (P34, patient with OG cancer) |
|  | Inadequate pain and fatigue management and its consequences | *“I mean, I couldn't even shower myself. I was out of breath. It was terrible. I had no one helping me... house cleaning and things like that. I couldn't do that for months on end.”* (P4, patient with pancreatic cancer)  *“(If I could) go and work, it would (be) terrific, it would be great, and I could probably earn more money, but I just can’t do it. I don’t know how I’m going to be, from one day to the next. Like, today I could be fine, I can go out, do something. The next day, I’ll just suffer, and just sit there all day, and not do a thing.”* (P18, patient with OG cancer) |
|  | Demanding role of caregivers | *“I appealed to My Aged Care for some domestic help…I was trying to, every couple of hours, get him to eat and do things, and then medications and blood level testings, insulin, it was - it was really quite full (on)”* (P13, caregiver of patient with pancreatic cancer) |
|  | Lack of continuity of care | *“…sometimes when I get sick, it’s like, I don’t know who I should sort of go to about my problem…am I still under the oncologist’s care even though the chemotherapy is finished? I’m not quite sure who I’m now meant to go to at this sort of stage.”* (P10, patient with pancreatic cancer). |
| Caregiver’s desire for greater support | Guidance for appropriately caring for patient | *“The hospital made no attempt to explain to me what I needed to do. I had to do basically a crash course in how to use the feed, how to feed her, how to look after her and I found that very difficult in the first week or so with working out exactly what I needed to do.”* (P26, caregiver of patient with pancreatic cancer) |
|  | Emotional support | *“My husband being the patient, obviously they were offering the counselling to him. If I was the doctor, I would have said, okay, you’ve got your wife, but I would have asked me the question, then, how are you coping with all this, you know?”* (P6, caregiver of patient with pancreatic cancer). |
|  | Financial support for regional-dwelling caregivers | *“The hospital didn’t offer any support in the way of accommodation or anything like that for partners.”* (P26, caregiver of patient with pancreatic cancer) |
| A multidisciplinary care team is the hallmark of a positive supportive care experience | Cancer care coordinator – a ‘one-stop-shop’ | *“It gave you relief inside, that you knew that it (cancer care coordinator) was there…and I wouldn’t go to the GP to get the support, I’d rather go – ring the liaison nurse, and I think she would have directed me anywhere I’ve got to go, for whatever problems I would have had.”* (P18, patient with OG cancer) |
|  | Palliative care – ‘a form of companionship’ | *“I initially misunderstood the value of these (palliative care) nurses, which is terrific, because they just pop in and see how you’re going, they check your blood pressure, anything that’s worrying us, and it’s sort of a form of companionship…”* (P19, patient with OG cancer)  *“It’s important for me because if I’m concerned about something, I have someone else to refer to…we can talk for an hour, which you can’t really do with your GP.”* (P20, caregiver of patient with OG cancer). |
|  | Community support services | *“I found Pancare on the internet…we did at one stage speak to the nurse there, who had a long chat with us and offered help if we needed it…that was something the hospital could have done. They could have given information on that early in the piece actually.”* (P26, caregiver of patient with pancreatic cancer) |
